# Accuracy is inaccurate: Why a focus on diagnostic accuracy for medical chatbot AIs will not lead to improved health outcomes

**DOI:** 10.1111/bioe.13365

**Published:** 2024-10-30

**Authors:** Stephen R. Milford

**Affiliations:** ^1^ Institute for Biomedical Ethics Basel University Basel Switzerland; ^2^ Department of Theology North‐West University Potchefstroom South Africa

**Keywords:** Chatbot AIs, ChatGPT, doctor–patient relationships, improved health outcomes, medical diagnosis

## Abstract

Since its launch in November 2022, ChatGPT has become a global phenomenon, sparking widespread public interest in chatbot artificial intelligences (AIs) generally. While not approved for medical use, it is capable of passing all three United States medical licensing exams and offers diagnostic accuracy comparable to a human doctor. It seems inevitable that it, and tools like it, are and will be used by the general public to provide medical diagnostic information or treatment plans. Before we are taken in by the promise of a golden age for chatbot medical AIs, it would be wise to consider the implications of using these tools as either supplements to, or substitutes for, human doctors. With the rise of publicly available chatbot AIs, there has been a keen focus on research into the diagnostic accuracy of these tools. This, however, has left a notable gap in our understanding of the implications for health outcomes of these tools. Diagnosis accuracy is only part of good health care. For example, crucial to positive health outcomes is the doctor–patient relationship. This paper challenges the recent focus on diagnostic accuracy by drawing attention to the causal relationship between doctor–patient relationships and health outcomes arguing that chatbot AIs may even hinder outcomes in numerous ways including subtracting the elements of perception and observation that are crucial to clinical consultations. The paper offers brief suggestions to improve chatbot medical AIs so as to positively impact health outcomes.

## 1 INTRODUCTION: THE RISE OF DR GPT

Natural Language Processing (NLP) artificial intelligence (AI) has been around since 1966,^1^ and over the years has significantly improved. Today one can access NLP applications by telephone, computer, and other digital platforms. However, it was not until the release of OpenAI's ChatGPT in late November 2022 that the public discovered the power of such chatbots.^2^ With over 100 million users within its first two months of launch, ChatGPT is the fastest‐growing consumer application of all time.^3^ In August 2023, its average monthly usage stood at 1.43 billion sessions—slightly down from its May highs of 1.8 billion, and boasts a market capitalisation of $90 billion.^4^ It has been specifically trained on conversational prompts to respond to a wide range of topics, most interestingly of which is its ability to respond to medical queries.^5^

That an AI tool may have medical uses is not, per se, novel. AI has been around since 1955,^6^ with the first computer‐aided detection tools for medical diagnosis being FDA approved in 1998.^7^ Today, AI technologies are already impacting health care with the promise of revolutionising the sector.^8^ AI has the ability to handle enormous amounts of structured as well as unstructured data—such as human speech, or hand‐written medical notes—and offers the promise of efficient clinical decisions by comparing data across billions of data points. In many ways, this may enable AI‐based applications to surpass human physicians at some tasks.^9^ Currently AI is instrumental in solving practical organisational challenges^10^; automating administrative work^11^ including producing medical notes^12^; predicting healthcare costs^13^; assessing mortality risks^14^; and even reducing ‘no‐shows’ for colonoscopies.^15^

More than this, AI is already present in direct patient care. This includes patient screening,^16^ triage,^17^ support in clinical decision‐making,^18^ and assisting in the diagnosis of numerous illnesses, from cancer^19^ to Covid‐19.^20^ So promising is this technology that the past few years have seen a ‘gold rush to purchasing AI tools’^21^ with millions being spent on AI centres for diagnosis and treatment.^22^

However, NLP tools such as ChatGPT are significantly different to technological medical tools such as ‘traditional’ symptom checkers. Symptom checkers have been available for some time, consider for example Ada, Bablyon, Buoy, and Your.MD. Such tools have demonstrated relatively accurate diagnosis and are considered a positive contribution to the medical profession.^23^ Yet, symptom checkers are limited, especially in their ability to offer follow‐up responses to user prompts. NLP chatbots such as ChatGPT, on the other hand, are far more intuitive, using natural conversations and user‐friendly interfaces. They produce a high level of concordance, understanding, and non‐obvious insight in their explanations.^24^ Furthermore, NLP tools are trained on vast amounts of data, in some cases live data, and incorporate an incredibly broad range of topics—much like a human doctor who is an agent with more than simply medical knowledge. If this were not enough, tools like ChatGPT are supported by far greater resources than many of the symptom checkers on the market today. With billions of dollars backing their development, it is no wonder they are evolving at an incredible rate.^25^

When all this is considered, it seems inevitable that tools like ChatGPT will be used by the general public for medical diagnosis.^26^ As Harvard Professor Mehrotra eloquently states: “The idea that we would tell patients they shouldn't use these tools seems implausible. They're going to use these tools.”^27^ It is conceivable that patients may use these tools as a first point of contact, or even instead of consulting with a human doctor. Naturally, this raises numerous ethical issues. What might the impact be if patients use these tools to supplement, or even substitute, human doctors? Is our focus on accuracy justified, or is there perhaps more to patient health outcomes than accurate diagnosis?

## 2 THE INACCURACY OF ACCURATE DIAGNOSIS

At first, it appears that the public's use of NLP tools for medical diagnosis may not be detrimental. Take ChatGPT for example. While not designed or approved for medical use, it is capable of providing medical information and diagnosis, including differential diagnosis.^28^ What is perhaps most striking is that this NLP tool is able to pass all three United States medical licensing exams^29^ as well as the national Italian residency admission exam.^30^ Furthermore, it is accurate in the diagnosis of a number of common diseases. For example, it achieves an overall accuracy of 94.1% in predicting common urinary diseases^31^ and an 85% accuracy in the provisional diagnosis of cornea eye disease.^32^ Recent tests on diagnostic accuracy have shown iterative improvements for ChatGPT 4.0 over its predecessor ChatGPT 3.5.^33^ To be fair, ChatGPT is not the only large language model that can be used for medical diagnosis. Google's Bard, GPT‐Neo, and MedBot are examples of strong competitors. Yet, ChatGPT consistently scores highly in comparative diagnostic tests.^34^

The accuracy of these NLP tools appears to be good news for global health care. Consider that the availability of human doctors is often in high demand and millions of people around the world have a severely curtailed ability to consult with a human doctor. In many contexts, traditional doctor‐heavy health care is simply not practical. For example, in remote regions of high‐income countries or some cities in low‐income contexts.^35^ NLP tools have the potential to reduce costs, alleviate pressure on overly burdened healthcare systems, and mitigate health disparities in many under‐served populations.

Even in high‐income countries, these tools may be incredibly helpful. For example, Switzerland (one of the richest countries in the world, with a high‐ranking healthcare system) is eager to digitise its healthcare.^36^ Health insurers in Switzerland are already incorporating AI into their businesses to improve patient experiences and reduce costs.^37^ It is conceivable that Swiss insurance companies may add AI assistance to their lower cost insurance plans in much the same way as they have added telehealth in recent years. Consequently, in the near future, even in rich countries like Switzerland, health insurance plans may require patients to first consult a chatbot before a human doctor as a way of reducing premiums.

Yet before we get carried away by the accuracy of these tools, it is worth cautioning that accurate diagnosis—while important—is not the sole contributor to good health outcomes. There are numerous aspects that need to be taken into consideration. These includes socio‐economic conditions, and even individual patient personalities.^38^ In particular, one could mention the significant influence of doctor–patient relationships. There is now ample evidence demonstrating that doctor–patient relationships have a causal link to positive health outcomes.^39^ A positive doctor–patient relationship improves patient activation,^40^ better adherence to treatment plans,^41^ improved treatment‐related behaviour,^42^ and a reduction in unnecessary prescriptions and interventions, such as antibiotics or futile interventions.^43^

Good doctor–patient relationships assist physicians to inquire and help alleviate patient's concerns, encourage physical activity, and set positive goals.^44^ This offers better emotional support and results in lower depression scores reported by patients.^45^ Of particular note is that a positive doctor–patient relationship has been shown to reduce the subjective symptom burden experienced by patients.^46^ In other words, patients experience less suffering when they partner positively with their health professional. This is the case even in palliative care, with end‐of‐life discussions being associated with higher quality of dying and a positive impact on the long‐term bereavement adjustment for patients’ family members.^47^

To be fair, there are significant advantages to new medical NLP tools. With more than 117 medical chatbot applications either released, or soon to be released,^48^ patients are already directly interacting with these tools on a regular basis.^49^ This includes chatbots that support general well‐being, for example, Emma 50 or Vivibot^51^; mental health, for example, Elomia 52 or Woebot^53^; and also chatbots that go so far as to provide digital counselling.^54^ Indeed, one of the world's largest companies, Nvidia, has recently announced AI‐powered healthcare ‘agents’ that reportedly outperform human nurses.^55^ While research is still in its infancy, there are some indications that AI tools like these might represent useful additions to promoting individualised care, especially for mental health and general well‐being.^56^

One significant additional benefit to medical NLP AI tools is patient education. It is well‐established that positive doctor–patient relationships can help improve education which itself has been shown to help patients gain control over complex diseases.^57^ Consequently, better education can lead to improved quality of life^58^ and a significant impact on patients’ health outcomes.^59^ Although it should be noted that patients who displayed in‐depth knowledge of their conditions were sometimes seen as non‐compliant by non‐specialist physicians, as these patients tended to challenge treatment decisions more often than less informed patients.^60^ In this particular regard, it may be argued that new NLP tools like ChatGPT can boost patient education. As patients ‘discuss’ their medical diagnosis with NLP AIs, they are afforded endless opportunity to ask questions and learn about their condition. This includes accessing the latest research and challenging the responses given by the AI. This process of ‘discussion’ could improve patient education if the information provided by the AI tool is accurate.

Notwithstanding the potential benefit of NLP tools to be used to supplement health care in areas such as mental health or to improve patient education, the role of the doctor–patient relationship cannot be underestimated: ‘the medical visit (i.e. the medical encounter) plays a pivotal role in the healthcare process. In fact, doctor–patient communication is one of the most essential dynamics in health care, affecting the course of patient care and patient compliance with recommendations for care.’^61^ It is, therefore, no surprise that the WHO has gone to great lengths to enact practices to strengthen doctor–patient relationships—especially in developing countries.^62^

An unhealthy obsession with diagnostic accuracy may offer a challenge to the work of organisations like the WHO that, over the last few decades, have sought to challenge paternalism, promote shared decision‐making, and strengthen doctor–patient relationships.^63^ Introducing AI into this relationship is sure to have an impact. Indeed, some are already arguing that the binary doctor–patient relationship is evolving to be a triad between patient, doctor, and AI.^64^ If arguments are true for some non‐linguistic‐based AI tools, how much more so for NLP tools that might offer high diagnostic accuracy along with the ability to discuss diagnoses in the comfort of one's home without the resource constraints imposed on healthcare institutions?

## 3 DIAGNOSTIC CHATBOTS MIGHT ACTUALLY IMPEDE HEALTH OUTCOMES

Globally, healthcare systems are under increased strain and patients are paying for this in numerous ways such as longer waiting times.^65^ NLP AI tools offer assistance by promising to provide an accurate diagnostic tool which is easy to deploy and relatively cheap. Very recent research into these tools has focused on their diagnostic accuracy, but not on their ability to positively influence health outcomes. The lack of research is understandable considering their new‐found popularity. Nevertheless, hyped headlines about AIs passing medical exams cloud the broader discussion on how to improve health outcomes.

Evidence suggests that health outcomes are generally favourably impacted when technological tools are implemented in health care.^66^ Patients and doctors agree that the introduction of the computer into the examination room has had positive effects. Nevertheless, there are concerns about the improper use of technology, its effects on doctor–patient relationships, and the knock‐on impact on health outcomes.^67^ In this sense, NLP tools, as technological advances deployed in healthcare settings, are not unique.

However, NLP tools such as ChatGPT, with their incredible popularity, ease of use, and widespread deployment, make this challenge even more pressing. There is now an urgent need to take up Córdova González's call to ‘deepen the understanding of the effects of the introduction of the computer [and AI] in the doctor‐patient setting.’^68^ In particular, there is a need to precisely identify the attributes of the doctor–patient relationship that positively influence health outcomes and to consider if (and how) it may be possible to incorporate these attributes into a well‐designed NLP tool that offers both accurate diagnosis and positively influences health outcomes.

There has already been some work to describe the attributes associated with health‐affirming doctor–patient relationships. For example, effective communication skills on both the doctor's and patient's side have been shown to be decisive.^69^ This includes the ability to build relationships (with empathy); gather, share, and provide information; reach agreement; and make shared decisions.^70^ While there is ample evidence to demonstrate the effectiveness of communication training for doctors,^71^ communication remains an incredibly complex phenomenon that includes not only verbal but also non‐verbal elements. Often it is these non‐verbal elements that are responsible for a disproportionate influence on doctor–patient communication.^72^

It is not just the lack of non‐verbal communication that is at stake, it is the very subtraction of the elements of perception and observation in clinical consultation that is problematic for AI tools. During consultations, healthcare professionals (HCPs) make use of their vision, hearing, touch, and smell to gather important diagnostic data. Consequently, the skills necessary for clinical observation are essential diagnostic tools. What is noteworthy is the one‐sided nature of the process of gathering this data. Patients do not necessarily volunteer such information. It is observed by the HCP who has undergone years of training to hone such observational skills (often in the form of ‘bedside’ training led by other HCPs). As new technologies, including AI technologies, have developed over the last few decades, these learned observational skills have declined amongst HCPs.^73^ The advent of advanced medical AI may exacerbate this decline. This in turn could disproportionately affect vulnerable members of society. For example, those with mental challenges; infants and young children; or those on the autism spectrum. Observational skills are vital for good healthcare amongst population groups such as these. It is these observational skills that are not available to an NLP AI—at least not at present.^74^

Apart from verbal and non‐verbal communication, trust is broadly considered a fundamental aspect of healthy doctor–patient relationships across diverse medical specialities.^75^ Trust, however, has been a particular concern in the context of AI generally. It has been shown that the public's ability to trust AI directly influences their acceptance and adoption of these technologies.^76^ Yet, engendering this trust is challenging and involves numerous aspects: from the characteristics of the AI, such as its performance; predictability; reliability, and so forth, to the particularities of users themselves, such as age; gender; culture; education, and so forth.^77^ Furthermore, explainability and transparency (both of which have been shown to be vital to human–AI trust in medical contexts) has proved difficult to achieve due to the opaque nature of AI decisions—the so‐called ‘black‐box’ problem.^78^

If NLP tools are to go beyond simply providing accurate diagnosis to having a positive impact on patient health outcomes, it is attributes such as these that need to be practically incorporated into their programming. Trust, open communication, and shared decision‐making must form the context in which their accurate diagnosis and treatment plans are given. Failure to incorporate these aspects into their design may actually impede health outcomes as these tools undermine the work done to strengthen doctor–patient relationships elsewhere. Yet incorporating these aspects is, understandably, no small challenge.

## 4 THE CHALLENGES OF HUMAN‐LIKE AI–PATIENT RELATIONSHIPS

To say that there are attributes to doctor–patient relationships that positively influence health outcomes and that these attributes must be incorporated into NLP programming, is not to underestimate the scale of the challenge. We may briefly point to some of these here. To begin, there are serious technical challenges to translating human relational values into a programme. It is well‐known that AI lacks empathy or compassion,^79^ and there may be serious issues in building trust with an AI. Apart from the black‐box challenge, tools such as ChatGPT make numerous errors in their responses, including fabricating academic references. What is perhaps more concerning is their tendency to repeat certain types of errors (such as making up references) even when a user draws attention to it.^80^

Apart from the technical hurdle of translating human relational values into AI programmes, engendering trust, and producing accurate results, it is the attributes of communication itself that is often complex, even for human doctors. For example, patients and doctors often miscommunicate.^81^ This could be because they each understand the patient's experience and situation differently^82^ or because the expectations of good communication differ depending on the context. There is evidence to support the argument that different medical specialities require different communicative strategies.^83^ Designing NLP tools for improved communication, therefore, may require NLP AIs to utilise different communicative strategies depending on the medical diagnosis, user, or general context. Distilling the exact communicative strategy required for each medical specialism, user, and context is a mammoth task, one we may never accomplish.

If that were not challenging enough, there is evidence to suggest that differences remain in the ‘constructions and perceptions of desirable and undesirable doctor attributes from region to region around the world.’^84^ Doctor–patient relationships are shaped by different cultures, social norms, and institutions. Designing NLP tools to take these attributes into consideration when answering a patient's health questions is a further challenge in itself.

Considering these challenges, it is possible that solutions do not lie only in modifying NLP tools and their programming, but in patients themselves and their contexts. For example, modifying patient promptings. The prompts given by NLP users are fundamental to accurate responses by NLP tools.^85^ The term ‘prompt engineering’ is relatively new in public discourse. When ChatGPT was launched, the public knew very little about this concept. Today, however, numerous courses are available to the public on how to properly prompt NLP AIs.^86^ Yet much of the public remain unaware that improving prompts can improve NLP responses—although awareness of this aspect is rapidly growing. Providing prompts that elicit not only accurate diagnosis but foster healthy patient—AI relationships is a big obstacle for patients’ use of NLP tools. Just as in building trust with a human doctor, in all likelihood, it is not only NLP tools that will need to be trained but patients themselves on how to properly prompt these tools.

## 5 CONCLUSION

NLP AI tools are rapidly advancing with each iteration improving on the next. Today readily available public tools such as ChatGPT are able to offer diagnostic accuracy akin to a human doctor. While this may present improvements on the current status quo in some contexts—such as where access to human doctors is severely curtailed—there are reasons to be cautious. Our discussions have argued that positive health outcomes depend on more than simply accurate diagnosis. In particular, there is ample evidence to suggest that doctor–patient relationships have a direct bearing on health outcomes. If NLP tools are used by patients for medical diagnosis and treatment plans, it is imperative that they are designed with more than just accuracy in mind. A way must be found to incorporate non‐verbal clinical observational data, as well as attributes such as respect, trust, and shared decision‐making into the broader context of their diagnostic programming. Failure to do so may undermine the important work undertaken over the last few decades to improve health outcomes. Consequently, it would be inaccurate to assume that accurate diagnosis will automatically lead to improved health outcomes.

## Footnotes

1 Weizenbaum, J. (1966). ELIZA—A computer program for the study of natural language communication between man and machine. *Communications of the ACM*, *9*(1), 36–45. https://doi.org/10.1145/365153.365168

2 Eysenbach, G. (2023). The role of ChatGPT, generative language models, and artificial intelligence in medical education: A conversation with ChatGPT and a call for papers,” *JMIR Medical Education*, *9*, e46885, https://doi.org/10.2196/46885

3 Baruffati, A. (2023, February 22). *ChatGPT statistics 2023 revealed: Insights & trends*. https://blog.gitnux.com/chat-gpt-statistics/

4 Nerdynav. (2023, October 1). *107 up‐to‐date ChatGPT statistics & user numbers*. https://nerdynav.com/chatgpt-statistics/

5 Gilson, A., Safranek, C. W., Huang, T., Socrates, V., Chi, L., Taylor, R. A., & Chartash, D. (2023). How does ChatGPT perform on the United States medical licensing examination? The implications of large language models for medical education and knowledge assessment. *JMIR Medical Education*, *9*, e45312. https://doi.org/10.2196/45312

6 Jaiswal, R., Ramesh Lal, S., Girish, K., Samiran, N. (2020). Artificial intelligence in medical diagnosis. *Current Medicine Research and Practice, 10*, 286, https://doi.org/10.4103/cmrp.cmrp_56_20

7 Elmore, J. G., & Lee, C. I. (2022). Artificial intelligence in medical imaging: Learning from past mistakes in mammography. *JAMA Health Forum*, *3*(2), e215207. https://doi.org/10.1001/jamahealthforum.2021.5207

8 Lebovitz, S., Lifshitz‐Assaf, H., & Levina, N. (2022). To engage or not to engage with AI for critical judgments: How professionals deal with opacity when using AI for medical diagnosis. *Organization Science*, *33*(1), 126–148. https://doi.org/10.1287/orsc.2021.1549

9 Jaiswal, R., et al., op. cit. note 6.

10 Kellogg, K. C., Valentine, M. A., & Christin, A. (2020). Algorithms at work: The new contested terrain of control. *Academy of Management Annals*, *14*(1), 366–410. https://doi.org/10.5465/annals.2018.0174

11 Brynjolfsson, E., & Mitchell, T. (2017). What can machine learning do? Workforce implications. *Science*, *358*(6370), 1530–1534. https://doi.org/10.1126/science.aap8062

12 Bala, S., Keniston, A., & Burden, M. (2020). Patient perception of plain‐language medical notes generated using artificial intelligence software: Pilot mixed‐methods study. *JMIR Formative Research*, *4*(6), e16670, https://doi.org/10.2196/16670

13 Kan, H. J., Kharrazi, H., Chang, H. Y., Bodycombe, D., Lemke, K., & Weiner, J. P. (2019). Exploring the use of machine learning for risk adjustment: A comparison of standard and penalized linear regression models in predicting health care costs in older adults. *PLoS One*, *14*(3), e0213258, https://doi.org/10.1371/journal.pone.0213258

14 Delahanty, R. J., Kaufman, D., & Jones, S. S. (2018). Development and evaluation of an automated machine learning algorithm for in‐hospital mortality risk adjustment among critical care patients. *Critical Care Medicine*, *46*(6), e481. https://doi.org/10.1097/CCM.0000000000003011

15 The Medical Futurist. (2021, August 31). The top 12 healthcare chatbots. *The Medical Futurist*. https://medicalfuturist.com/top-12-health-chatbots/

16 Håvik, R., Wake, J. D., Flobak, E., Lundervold, A., & Guribye, F. (2019). A conversational interface for self‐screening for ADHD in adults. In S. S. Bodrunova, O. Koltsova, A. Følstad, H. Halpin, P. Kolozaridi, L. Yuldashev, A. Smoliarova, & H. Niedermayer (Eds.), *Internet science*. Lecture Notes in Computer Science (pp. 133–144). Springer International Publishing. https://doi.org/10.1007/978-3-030-17705-8_12

17 Chang, P., Sheng, Y.‐H., Sang, Y.‐Y., & Wang, D. W. (2008). Developing a wireless speech‐ and touch‐based intelligent comprehensive triage support system,” *CIN: Computers, Informatics, Nursing*, *26*(1), 31. https://doi.org/10.1097/01.NCN.0000304754.49116.b4

18 Garcia‐Vidal, C., Sanjuan, G., Puerta‐Alcalde, P., Moreno‐García, E., & Soriano, A. (2019). Artificial intelligence to support clinical decision‐making processes. *EBioMedicine*, *46* 27–29. https://doi.org/10.1016/j.ebiom.2019.07.019

19 Savage, N. (2020). How AI is improving cancer diagnostics. *Nature*, *579*(7800), S14–S16. https://doi.org/10.1038/d41586-020-00847-2

20 Benameur, N., Mahmoudi, R., Zaid, S., Arous, Y., Hmida, B., & Bedoui, M. H. (2021). Sars‐Cov‐2 diagnosis using medical imaging techniques and artificial intelligence: A review. *Clinical Imaging*, *76*, 6–14. https://doi.org/10.1016/j.clinimag.2021.01.019

21 Lebovitz, S., et al., op. cit. note 8.

22 O'Dowd, A. (2019). Government pins hopes on £250 m AI centre for faster diagnosis and treatment. *British Medical Journal*, *366*, 1.

23 Ćirković, A. (2020). Evaluation of four artificial intelligence‐assisted self‐diagnosis apps on three diagnoses: Two‐year follow‐up study. *Journal of Medical Internet Research*, *22*(12), e18097. https://doi.org/10.2196/18097

24 Kung, T. H., Cheatham, M., Medenilla, A., Sillos, C., De Leon, L., Elepaño, C., Madriaga, M., Aggabao, R., Diaz‐Candido, G., Maningo, J., & Tseng, V. (2023). Performance of ChatGPT on USMLE: Potential for AI‐assisted medical education using large language models. *PLOS Digital Health*, *2*(2), e0000198. https://doi.org/10.1371/journal.pdig.0000198

25 Q.ai. (2023). *Microsoft confirms its $10 billion investment into ChatGPT, changing how Microsoft competes with Google, Apple and other tech giants*. Forbes. https://www.forbes.com/sites/qai/2023/01/27/microsoft-confirms-its-10-billion-investment-into-chatgpt-changing-how-microsoft-competes-with-google-apple-and-other-tech-giants/

26 Moore, S. (2023, March 27). *What does ChatGPT mean for healthcare?* News‐Medical.net. https://www.news-medical.net/health/What-does-ChatGPT-mean-for-Healthcare.aspx

27 Weintraub, K. (2023, February 2). ChatGPT is poised to upend medical information. For better and worse. *USA TODAY*. https://www.usatoday.com/story/news/health/2023/02/26/chatgpt-medical-care-doctors/11253952002/

28 Garg, A. (2022, December 27). *What is ChatGPT, and its possible use cases?* Net solutions. https://www.netsolutions.com/insights/what-is-chatgpt/

29 Gilson, A., et al., op. cit. note 5; Kung, T. H., et al., op. cit. note 24.

30 Bonetti, M. A., Giorgino, R., Afflitto, G. G., De Lorenzi, F., & Egro, F. M. (2023). How does ChatGPT perform on the Italian residency admission national exam compared to 15,869 medical graduates? *Annals of Biomedical Engineering*, *52*, 745–749. https://doi.org/10.1007/s10439-023-03318-7

31 Xv, Y., Peng, C., Wei, Z., Liao, F., & Xiao, M. (2023). Can Chat‐GPT a substitute for urological resident physician in diagnosing diseases?: A preliminary conclusion from an exploratory investigation. *World Journal of Urology*, *41*(9), 2569–2571. https://doi.org/10.1007/s00345-023-04539-0

32 Delsoz, M., Madadi, Y., Munir, W. M., Tamm, B., Mehravaran, S., Soleimani, M., Djalilian, A., & Yousefi, S. (2023, August 28) Performance of ChatGPT in diagnosis of corneal eye diseases. *medRxiv*, 2023.08.25.23294635. https://doi.org/10.1101/2023.08.25.23294635

33 Shieh, A., Tran, B., He, G., Kumar M., Freed, J. A., & Majety, P. (2024, April 23). Assessing ChatGPT 4.0's test performance and clinical diagnostic accuracy on USMLE STEP 2 CK and clinical case reports. *Scientific Reports*, *14*, 9330. https://doi.org/10.1038/s41598-024-58760-x

34 Caruccio, L., Cirillo, S., Polese, G., Solimando, G., Sundaramurthy, S., & Tortora, G. (2024). Can ChatGPT provide intelligent diagnoses? A comparative study between predictive models and ChatGPT to define a new medical diagnostic bot. *Expert Systems With Applications*, *235*, 121186. https://doi.org/10.1016/j.eswa.2023.121186

35 Guo, J., & Li, B. (2018). The application of medical artificial intelligence technology in rural areas of developing countries. *Health Equity*, *2*(1), 174–181. https://doi.org/10.1089/heq.2018.0037; van Veen, T., Binz, S., Muminovic, M., Chaudhry, K., Rose, K., Calo, S., Rammal, J.‐A., France, J., & Miller, J. B. (2019). Potential of mobile health technology to reduce health disparities in underserved communities. *The Western Journal of Emergency Medicine*, *20*(5), 799–802. https://doi.org/10.5811/westjem.2019.6.41911

36 Hämmerli, M., Müller, T., Biesdorf, S., Ramezani, S., Sartori, V., Steinmann, M., Van der Veken, L., Fleisch, E., & von Wangenheim, F. (2021). *Digitalisierung Im Gesundheitswesen: Die 8,2‐Mrd.‐Chf‐Chance Für Die Schweiz*. McKinsey Digital.

37 SWICA. (2022). *Many different reasons to choose SWICA insurance—SWICA*. https://www.swica.ch/en/private/why-swica/swica-advantages/advantages; Walder, B. (2022). *Diese Grossen Krankenkassen Bieten Den Besten Service*. K‐Tipp. https://www.ktipp.ch/artikel/artikeldetail/diese-grossen-krankenkassen-bieten-den-besten-service

38 Atlas, L., S., & Zweig, R. (2019). The doctor‐patient relationship, personality, mood, and functioning in older adults. *Innovation in Aging*, *3*(Supplement_1 Program Abstracts from the GSA 2019 Annual Scientific Meeting “Strength in Age—Harnessing the Power of Networks), S161–S162. https://doi.org/10.1093/geroni/igz038.578

39 Chipidza, F. E., Wallwork, R. S., & Stern, T. A. (2015). Impact of the doctor‐patient relationship. *The Primary Care Companion for CNS Disorders*, *17*(5), 27354. https://doi.org/10.4088/PCC.15f01840; Riedl, D., & Schüßler, G. (2017). The influence of doctor‐patient communication on health outcomes: A systematic review. *Zeitschrift Für Psychosomatische Medizin Und Psychotherapie*, *63*(2), 131–150. https://doi.org/10.13109/zptm.2017.63.2.131; Stewart, M. A., McWhinney, I. R., & Buck, C. W. (1979). The doctor/patient relationship and its effect upon outcome. *The Journal of the Royal College of General Practitioners*, *29*(199), 77–82.

40 Alexander, J. A., Hearld, L. R., Mittler, J. N., & Harvey, J. (2012). Patient‐physician role relationships and patient activation among individuals with chronic illness. *Health Services Research*, *47*(3pt1), 1201–1223. https://doi.org/10.1111/j.1475-6773.2011.01354.x

41 Mahmoudian, A., Zamani, A., Tavakoli, N., Farajzadegan, Z., & Fathollahi‐Dehkordi, F. (2017). Medication adherence in patients with hypertension: Does satisfaction with doctor‐patient relationship work? *Journal of Research in Medical Sciences: The Official Journal of Isfahan University of Medical Sciences*, *22*, 48. https://doi.org/10.4103/jrms.JRMS_205_16; van Ganse, E. (2003). Factors affecting adherence to asthma treatment: Patient and physician perspectives. *Primary Care Respiratory Journal: Journal of the General Practice Airways Group*, *12*(2), 46–51, https://doi.org/10.1038/pcrj.2003.17

42 Parchman, M. L., Zeber, J. E., & Palmer, R. F. (2010). Participatory decision making, patient activation, medication adherence, and intermediate clinical outcomes in type 2 diabetes: A starnet study. *Annals of Family Medicine*, *8*(5), 410–417. https://doi.org/10.1370/afm.1161

43 Cals, J. W., Butler, C. C., Hopstaken, R. M., Hood, K., & Dinant, G. J. (2009). Effect of point of care testing for c reactive protein and training in communication skills on antibiotic use in lower respiratory tract infections: Cluster randomised trial. *BMJ (Clinical Research Ed.)*, *338*, b1374. https://doi.org/10.1136/bmj.b1374

44 Cabana, M. D., Slish, K. K., Evans, D., Mellins, R. B., Brown, R. W., Lin, X., Kaciroti, N., & Clark, N. M. (2014). Impact of physician asthma care education on patient outcomes. *Health Education & Behavior*, *41*(5), 509–517. https://doi.org/10.1177/1090198114547510

45 Fujimori, M., Shirai, Y., Asai, M., Kubota, K., Katsumata, N., & Uchitomi, Y. (2014). Effect of communication skills training program for oncologists based on patient preferences for communication when receiving bad news: A randomized controlled trial. *Journal of Clinical Oncology: Official Journal of the American Society of Clinical Oncology*, *32*(20), 2166–2172. https://doi.org/10.1200/JCO.2013.51.2756

46 Little, P., Everitt, H., Williamson, I., Warner, G., Moore, M., Gould, C., Ferrier, K., & Payne, S. (2001). Observational study of effect of patient centredness and positive approach on outcomes of general practice consultations. *BMJ (Clinical Research Ed.)*, *323*(7318), 908–911. https://doi.org/10.1136/bmj.323.7318.908

47 Wright, A. A., Zhang, B., Ray, A., Mack, J. W., Trice, E., Balboni, T., Mitchell, S. L., Jackson, V. A., Block, S. D., Maciejewski, P. K., & Prigerson, H. G. (2008). Associations between end‐of‐life discussions, patient mental health, medical care near death, and caregiver bereavement adjustment. *JAMA*, *300*(14), 1665–1673. https://doi.org/10.1001/jama.300.14.1665; Zhang, B., Wright, A. A., Huskamp, H. A., Nilsson, M. E., Maciejewski, M. L., Earle, C. C., Block, S. D., Maciejewski, P. K., & Prigerson, H. G. (2009). Health care costs in the last week of life: Associations with end‐of‐life conversations. *Archives of Internal Medicine*, *169*(5), 480–488. https://doi.org/10.1001/archinternmed.2008.587

48 Tracxn. (2024, January 10). *Mental health needs have multiplied: Support hasn't. Until now*.*Top Healthcare Chatbots Startups*. https://tracxn.com/d/trending-themes/startups-in-healthcare-chatbots/__AXRhOxVhBdHrB3kNRRV-rJNbuz66moUFeot5q0tnlG0

49 Abd‐Alrazaq, A. A., Alajlani, M., Ali, N., Denecke, K., Bewick, B. M., & Househ, M. (2021). Perceptions and opinions of patients about mental health chatbots: Scoping review. *Journal of Medical Internet Research*, *23*(1), e17828. https://doi.org/10.2196/17828; Sheth, A., Yip, H. Y., & Shekarpour, S. (2019). Extending Patient‐chatbot experience with internet‐of‐things and background knowledge: Case studies with healthcare applications. *IEEE Intelligent Systems*, *34*(4), 24–30. https://doi.org/10.1109/MIS.2019.2905748; Webster, P. (2023). Medical AI chatbots: Are they safe to talk to patients? *Nature Medicine*, *29*(11), 2677–2679. https://doi.org/10.1038/s41591-023-02535-w

50 Ghandeharioun, A., McDuff, D., Czerwinski, M., & Rowan, K. (2019, July 22). *EMMA: An emotion‐aware wellbeing chatbot*. arXiv. https://doi.org/10.48550/arXiv.1812.11423

51 Greer, S., Ramo, D., Chang, Y. J., Fu, M., Moskowitz, J., & Haritatos, J. (2019). Use of the chatbot ‘Vivibot’ to deliver positive psychology skills and promote well‐being among young people after cancer treatment: Randomized controlled feasibility trial. *JMIR mHealth and uHealth*, *7*(10), e15018. https://doi.org/10.2196/15018

52 Elomia. *Elomia health—Mental health chatbot*. Retrieved February 26, 2024, from https://elomia.com/

53 Woebot Health. Retrieved February 26, 2024, from https://woebothealth.com/

54 Cameron, G., Cameron, D. M. Megaw, G., Bond, R. B., Mulvenna, M., O ' Neill, S., Armour, C., Mctear, M. F. (2017). *Towards a Chatbot for digital counselling*. Proceedings of the 31st International BCS Human Computer Interaction Conference (HCI 2017), BCS Learning & Development. https://doi.org/10.14236/ewic/HCI2017.24; Inkster, B., Sarda, S., & Subramanian, V. (2018). An empathy‐driven, conversational artificial intelligence agent (Wysa) for digital mental well‐being: Real‐world data evaluation mixed‐methods study. *JMIR mHealth and uHealth*, *6*(11), e12106. https://doi.org/10.2196/12106

55 Dumas, B. (2024, March 21). *Nvidia announces AI‐powered health care ‘agents’ that outperform nurses—And cost $9 an hour*. Text.Article, FOXBusiness (Fox Business. https://www.foxbusiness.com/technology/nvidia-announces-ai-powered-health-care-agents-outperform-nurses-cost-9-hour

56 Boucher, E. M., Harake, N. R., Ward, H. E., Stoeckl, S. E., Vargas, J., Minkel, J., Parks, A. C., & Zilca, R. (2021). Artificially intelligent chatbots in digital mental health interventions: A review. *Expert Review of Medical Devices*, *18*(Suppl. 1), 37–49. https://doi.org/10.1080/17434440.2021.2013200

57 Snow, R., Humphrey, C., & Sandall, J. (2013). What happens when patients know more than their doctors? Experiences of health interactions after diabetes patient education: A qualitative patient‐led study. *BMJ Open*, *3*(11), e003583. https://doi.org/10.1136/bmjopen-2013-003583; Sperl‐Hillen, J., Beaton, S., Fernandes, O., Von Worley, A., Vazquez‐Benitez, G., Parker, E., Hanson, A., Lavin‐Tompkins, J., Glasrud, P., Davis, H., Adams, K., Parsons, W., & Spain, C. V. (2011). Comparative effectiveness of patient education methods for type 2 diabetes: A randomized controlled trial. *Archives of Internal Medicine*, *171*(22), 2001–2010. https://doi.org/10.1001/archinternmed.2011.507

58 Aiarzaguena, J. M., Grandes, G., Gaminde, I., Salazar, A., Sánchez, A., & Ariño, J. (2007). A randomized controlled clinical trial of a psychosocial and communication intervention carried out by GPs for patients with medically unexplained symptoms. *Psychological Medicine*, *37*(2), 283–294. https://doi.org/10.1017/S0033291706009536; Martínez‐Moragón, E., Palop, M., de Diego, A., Serra, J., Pellicer, C., Casán, P., Caloto, M. T., Nocea, G., & ASMACOST Study Group. (2014). Factors affecting quality of life of asthma patients in Spain: The importance of patient education. *Allergologia Et Immunopathologia*, *42*(5), 476–484. https://doi.org/10.1016/j.aller.2013.06.006

59 Cabana, M. D., et al., op. cit. note 44.

60 Humphrey & Sandall, op. cit. note 57.

61 Matusitz, J., & Spear, J. (2014). Effective doctor‐patient communication: An updated examination. *Social Work in Public Health*, *29*(3), 252. https://doi.org/10.1080/19371918.2013.776416

62 WHO. (2013). *Strengthening the doctor‐patient relationship*. World Health Organization. Regional Office for South‐East Asia. https://iris.who.int/handle/10665/205942

63 Charles, C., Gafni, A., & Whelan, T. (1997). Shared decision‐making in the medical encounter: What does it mean? (or it takes at least two to tango). *Social Science & Medicine*, *44*(5), 681–692, https://doi.org/10.1016/s0277-9536(96)00221-3; Gulbrandsen, P. (2020). Shared decision making: Improving doctor‐patient communication. *BMJ*, *368*, m97–m97. https://doi.org/10.1136/bmj.m97

64 Lorenzini, G., Arbelaez Ossa, L., Shaw, D. M., & Elger, B. S. (2023). Artificial intelligence and the doctor–patient relationship expanding the paradigm of shared decision making. *Bioethics*, *37*(5). https://doi.org/10.1111/bioe.13158

65 McIntyre, D., & Chow, C. K. (2020). Waiting time as an indicator for health services under strain: A narrative review. *INQUIRY: The Journal of Health Care Organization, Provision, and Financing*, *57*, 0046958020910305. https://doi.org/10.1177/0046958020910305

66 Carini, E., Villani, L., Pezzullo, A. M., Gentili, A., Barbara, A., Ricciardi, W., & Boccia, S. (2021). The impact of digital patient portals on health outcomes, system efficiency, and patient attitudes: Updated systematic literature review. *Journal of Medical Internet Research*, *23*(9), e26189. https://doi.org/10.2196/26189

67 González, G. A. C. (2022). Electronic health records: Its effects on the doctor‐patient relationship and the role of the computer in the clinical setting. *Health and Technology*, *12*(2), 305–311. https://doi.org/10.1007/s12553-021-00634-7

68 Ibid: 305.

69 Setlhare, V., & Madiba, S. (2023). Doctor attributes that patients desire during consultation: The perspectives of doctors and patients in primary health care in Botswana. *Healthcare*, *11*(840), 840. https://doi.org/10.3390/healthcare11060840

70 Di Blasi, Z., Harkness, E., Ernst, E., Georgiou, A., & Kleijnen, J. (2001). Influence of context effects on health outcomes: A systematic review. *Lancet (London, England)*, *357*(9258), 757–762. https://doi.org/10.1016/s0140-6736(00)04169-6

71 Girgis, A., Cockburn, J., Butow, P., Bowman, D., Schofield, P., Stojanovski, E., D'Este, C., Tattersall, M. H., Doran, C., & Turner, J. (2009). Improving patient emotional functioning and psychological morbidity: Evaluation of a consultation skills training program for oncologists. *Patient Education and Counseling*, *77*(3), 456–462. https://doi.org/10.1016/j.pec.2009.09.018

72 Bommier, C., Mamzer, M. F., Desmarchelier, D., & Hervé, C. (2014). How nonverbal communication shapes doctor‐patient relationship: From paternalism to the ethics of care in oncology. *Journal International de Bioéthique*, *25*(4), 29. https://doi.org/10.3917/jib.254.0029

73 Braverman, I. M. (2011). To see or not to see: How visual training can improve observational skills. *Clinics in Dermatology*, Current Views on Contact Dermatitis, *29*(3), 343–346. https://doi.org/10.1016/j.clindermatol.2010.08.001

74 It should be noted that there is a move to incorporate medical smart devices, wireless sensors, and systems into NLP tools, Hemanth, D. J., Anitha, J., & Tsihrintzis, G. A. (Eds.). (2021). *Internet of medical things: Remote healthcare systems and applications*. Internet of Things. Springer International Publishing. https://doi.org/10.1007/978-3-030-63937-2. It is, at least theoretically, conceivable that the integration of certain connected devices such as cameras or smart wearables (watches, pacemakers, blood pressure monitors, etc.) might provide some of the attributes derived from clinical observations.

75 Anderson, S. R., Gianola, M., Medina, N., Perry, J. M., Wager, T. D., Losin, E. A. R. (2023). Doctor trustworthiness influences pain and its neural correlates in virtual medical interactions. *Cerebral Cortex (New York, N.Y.: 1991)*, *33*(7), 3421–3436. https://doi.org/10.1093/cercor/bhac281; Quigley, D. D., Elliott, M. N., Farley, D. O., Burkhart, Q., Skootsky, S. A., & Hays, R. D. (2014). Specialties differ in which aspects of doctor communication predict overall physician ratings. *Journal of General Internal Medicine*, *29*(3), 447–454. https://doi.org/10.1007/s11606-013-2663-2

76 Choung, H., David, P., & Ross, A. (2023). Trust in AI and its role in the acceptance of AI technologies. *International Journal of Human–Computer Interaction*, *39*(9), 1727–1739. https://doi.org/10.1080/10447318.2022.2050543

77 Kaplan, A. D., Kessler, T. T., Brill, J. C., & Hancock, P. A. (2023). Trust in artificial intelligence: Meta‐analytic findings. *Human Factors*, *65*(2), 337–359. https://doi.org/10.1177/00187208211013988

78 Quinn, T. P., Jacobs, S., Senadeera, M., Le, V., & Coghlan, S. (2022). The three ghosts of medical AI: Can the black‐box present deliver? *Artificial Intelligence in Medicine*, 124, 102158. https://doi.org/10.1016/j.artmed.2021.102158

79 Montemayor, C., Halpern, J., & Fairweather, A. (2022). In principle obstacles for empathic AI: Why we can't replace human empathy in healthcare. *AI & SOCIETY*, *37*(4), 1353–1359. https://doi.org/10.1007/s00146-021-01230-z; Kerasidou, A. (2020). Artificial intelligence and the ongoing need for empathy, compassion and trust in healthcare. *Bulletin of the World Health Organization*, *98*(4), 245–250. https://doi.org/10.2471/BLT.19.237198

80 Eysenbach, op. cit. note 2.

81 Sparks, L., & Villagran, M. (2010). *Patient and provider interaction: A global health communication perspective*. Polity Press.

82 Matthew BA Adamson et al. (2018). The doctor–patient relationship and information‐seeking behavior: Four orientations to cancer communication. *Journal of Palliative Care*, *33*(2), 79–87. https://doi.org/10.1177/0825859718759881

83 Quigley, D. D., et al., op. cit. note 75.

84 Setlhare & Madiba, op. cit. note 69.

85 Kung, T. H., et al., op. cit. note 24.

86 Vogel, M. (2024, May 27). *The 10 best free prompt engineering courses & resources for Chatgpt, Midjourney & Co*. Medium (blog). https://medium.com/@maximilian.vogel/the-10-best-free-prompt-engineering-courses-resources-for-chatgpt-midjourney-co-dd1865d4ad13

